# Early Detection of Atrial Fibrillation in Chronic Obstructive Pulmonary Disease Patients

**DOI:** 10.3390/medicina60030352

**Published:** 2024-02-20

**Authors:** Stanislav Kotlyarov, Alexander Lyubavin

**Affiliations:** 1 Department of Nursing, Ryazan State Medical University, 390026 Ryazan, Russia; 2 Lipetsk City Hospital №4, 398006 Lipetsk, Russia

**Keywords:** atrial fibrillation, chronic obstructive pulmonary disease, early diagnosis, comorbid course

## Abstract

Atrial fibrillation (AF) is an important medical problem, as it significantly affects patients’ quality of life and prognosis. AF often complicates the course of chronic obstructive pulmonary disease (COPD), a widespread disease with heavy economic and social burdens. A growing body of evidence suggests multiple links between COPD and AF. This review considers the common pathogenetic mechanisms (chronic hypoxia, persistent inflammation, endothelial dysfunction, and myocardial remodeling) of these diseases and describes the main risk factors for the development of AF in patients with COPD. The most effective models based on clinical, laboratory, and functional indices are also described, which enable the identification of patients suffering from COPD with a high risk of AF development. Thus, AF in COPD patients is a frequent problem, and the search for new tools to identify patients at a high risk of AF among COPD patients remains an urgent medical problem.

## 1. Introduction

About 8% of patients with chronic obstructive pulmonary disease (COPD) have atrial fibrillation (AF). At the same time, approximately 13% of patients with AF also have COPD [1,2]. The combination of these diseases triggers processes leading to the development of pulmonary hypertension, valvular insufficiency, and ventricular dilatation, which contributes to the progression of cardiovascular and lung diseases. Factors such as chronic hypoxia, persistent inflammation, endothelial dysfunction, myocardial remodeling, and the administration of β2-adrenergic receptor agonists significantly contribute to the pathogenesis of AF in patients with COPD. Despite the fact that the mechanisms of AF development are well studied, the possibilities for predicting the development of AF in patients with COPD are not fully understood. The effectiveness of the currently known methods for the early detection of AF in the COPD population also remains an open question. In this review, we will consider the prospects of the application of various methods for the early diagnosis of AF in COPD patients.

## 2. Impact of COPD on the Progression of Cardiovascular Disease

The course of COPD is characterized by the development of specific structural and functional changes in the cardiovascular system due to the direct or indirect effects of a number of harmful factors [3]. COPD is characterized by the involvement of various immune mechanisms and the development of local and systemic inflammation [4,5]. The depletion of the reserves of adaptive mechanisms leads to the initiation of pathological processes, which create a substrate for the development of atrial fibrillation. According to the Swedish registry (1995–2008), the presence of atrial fibrillation significantly increases mortality from all causes. This is especially true for patients with first-ever atrial fibrillation. According to the study, comorbidities, such as neoplasms, chronic kidney disease (CKD), and COPD, contributed significantly to an unfavorable prognosis [6]. On the other hand, known cardiovascular disease (CVD) risk factors, such as arterial hypertension (AHT), dyslipidemia, CKD, and obesity, are quite common in COPD patients [7]. To assess the risk for developing coronary heart disease (CHD), cerebrovascular disease, and diabetes mellitus in patients with COPD, Feary, J.R. analyzed data from 1,204,100 patients over 35 years of age. COPD was significantly associated with an increased risk of cardiovascular disease (odds ratio (OR): 4.98; 95% confidence interval (CI): 4.85–5.81; *p* < 0.001) and stroke (OR: 3.34; 95% CI: 3.21–3.48; *p* < 0.001) [8].

The population of patients suffering from the combination of COPD and AF is characterized by a high mortality rate. However, the contribution of COPD to cause-of-death statistics is underestimated because patients with COPD often die from CVD. The impacts of COPD in patients with AF on clinical outcomes, such as death from any cause and death from CVD, were evaluated in a study of 1975 participants divided into 2 groups: AF with COPD and AF without COPD. During the 1-year follow-up, all-cause death and the incidence of cardiovascular complications were significantly higher in the AF with COPD group (26.9% vs. 12.3%; *p* < 0.001 and 25.6% vs. 19.1%; *p* = 0.027, respectively), despite a comparable incidence of stroke in both groups (7.9% vs. 7.4%; *p* = 0.788) [9]. A large study conducted in Denmark from 1999 to 2018 was designed to assess all-cause mortality in patients with combined COPD and AF. Participants (62,806 patients; median age 75.0 years; 56.5% men) were divided into 3 groups according to the order of the diagnosis: patients in whom AF manifested before the diagnosis of COPD, patients in whom COPD was diagnosed before the onset of AF, and patients in whom COPD and AF were diagnosed simultaneously within 6 months. After 5 years of follow-up, 31,494 subjects (50.1%) had died. After adjustments for age, sex, the presence of type 2 diabetes mellitus, and histories of acute myocardial infarction, arterial hypertension, chronic heart failure (CHF), dyslipidemia, cancer, chronic kidney disease, and stroke, mortality was the highest in group 2 (hazard ratio (HR): 1.30; 95% CI: 1.27–1.33; *p* < 0.001) and group three (OR: 1.19; 95% CI: 1.16–1.23; *p* < 0.001), further demonstrating that the earlier COPD is diagnosed, the worse the long-term prognosis is [1].

The EORP-AF study also evaluated the impact of COPD on the prognosis of patients with AF. As in the studies presented above, patients with COPD had more CVD risk factors and comorbidities, including diabetes mellitus and CHF (*p* < 0.0001). After 1 year of follow-up, patients with AF and COPD had a higher risk of both CVD and all-cause mortality (*p* < 0.0001). Patients with COPD and AF were also more likely to achieve the combined endpoint of a thromboembolic event, bleeding, and cardiovascular death (*p* = 0.0001). Cox regression analysis showed that COPD was independently associated with an increased risk of all-cause mortality (OR: 1.55; 95% CI: 1.05–2.28; *p* = 0.0269). Thus, COPD in patients with AF was associated with higher rates of cardiovascular mortality, all-cause mortality, and the combined outcome of any thromboembolic event, bleeding, or cardiovascular death [10,11].

One recently published meta-analysis including 7 studies also showed a higher likelihood of a number of both thromboembolic and hemorrhagic complications in patients with a combination of AF and COPD. Thus, the HRs assessing the impacts of COPD on total and cardiovascular mortalities were 1.70 (95% CI: from 1.47 to 1.97; *p* < 0.0001) and 1.80 (95% CI: from 1.29 to 2.52; *p* = 0.0005), respectively. The incidence of hemorrhagic complications was also significantly higher in patients with AF and COPD (odds ratio (OR): 1.84; 95% CI: 1.58–2.14; *p* < 0.00001) [10].

## 3. Factors Associated with Developing Atrial Fibrillation in COPD Patients

The results of two meta-analyses clearly demonstrate the high risk for developing atrial fibrillation in patients with COPD. The first, which included 14 studies, examined the risk for developing AF in patients with COPD and asthma. The results showed that COPD (OR = 1.74; 95% CI: 1.70–1.79) and asthma (OR = 1.08; 95% CI: 1.04–1.12) were significantly associated with an increased risk of AF [12]. Twenty-one studies were selected for the second meta-analysis. The presence of COPD was significantly associated with the risk for developing AF (OR = 1.99; 95% CI: 1.46–2.70), ventricular arrhythmias (OR = 2.01; 95% CI: 1.42–2.85), and sudden cardiac death (OR = 1.68; 95% CI: 1.28–2.21) [12]. A number of pathogenetic features of COPD are currently known to contribute to the development and progression of the disease.

### 3.1. Decline in Pulmonary Function and Hypoxia

The most important manifestation of COPD is impaired lung function, which is manifested by a decrease in the respiratory flow rate with the development of chronic hypoxia. Thus, in the Takahata study conducted in Japan on 2917 patients with COPD, lung function indices were significantly worse in patients with AF than in patients without AF, regardless of sex, age, and the presence of CHF, and the forced expiratory volume in one second (FEV1) was an independent risk factor for the development of AF in patients with COPD [13]. Chronic hypoxia is a significant risk factor not only for AF but also for other cardiovascular diseases (CVDs). In a study by Hozawa, A., 13,842 initially intact patients with ischemic stroke (IS) and no evidence of CHD were followed for 13 years. During the follow-up period, 472 cases of IS were recorded. The study showed significant associations between reduced FEV1 and forced vital capacity (FVC) and the development of IS (*p* < 0.01) [14]. 

The associations between the severity of the bronchial obstruction, pulmonary hypertension, and the risk of AF were demonstrated in a small study of patients with normal left ventricular function. A reduced FEV1 was more frequently associated with chronic AF (*p* = 0.003; 95% CI: 0.963–0.993) and led to heart failure with a preserved left ventricular ejection fraction (EF) [15]. Another study involving 13,430 patients with a follow-up period of 13 years showed a 1.8-fold increase in the odds for developing AF in the group of patients with an FEV1 of less than 80% of normal compared with the group of patients with an FEV1 of greater than 80% of normal. In the group of patients with an FEV1 of less than 60% of normal, there was an 8-fold increase in the odds for developing AF compared to the control group [16]. Similar data were obtained in another study that included 2917 patients who were evaluated for lung function. The risk for developing AF was higher in patients with an FVC of less than 50% and an FEV1 of less than 50% and was independent of sex, patient age, the presence of left ventricular hypertrophy, and NT-pro-BNP levels [13]. The association of AF with the severity of the bronchial obstruction was demonstrated in a small study conducted in 2016. In it, the frequency of AF paroxysms correlated with FEV1 (r = −0.348; *p* = 0.013) and low blood oxygen saturation (r = −0.356; *p* = 0.011). A decline in FEV1 was also correlated with the left atrial volume index (χ2 = 7.0; *p* = 0.008) and left ventricular isovolumic relaxation time (IVRT) (χ2 = 7.9; *p* = 0.005) [17]. 

The relationship between atrial fibrillation and the severity of the bronchial obstruction was investigated in 80 patients with persistent AF, preserved left ventricular function, and COPD. Patients with a declining FEV1 and FEV1/FVC and an increased pulmonary artery pressure (PAP) had a higher incidence of COPD. A declining FEV1 was associated with a persistent form of AF (*p* = 0.003; 95% CI: 0.963–0.993) [15].

The association of AF with severe COPD in oxygen-dependent patients was investigated in a retrospective analysis of case histories from 2003 to 2014. Data from 1,345,270 patients were included in the study, of whom 244,488 (18.2%) had AF. The prevalence of AF increased from 12.9% in 2003 to 21.3% in 2014 (*p* < 0.0001). AF was a predictor of the risk of in-hospital death (OR: 1.54; 95% CI: 1.45–1.65), acute respiratory failure (OR: 1.09; 95% CI: 1.06–1.12), invasive mechanical ventilation (OR: 1.37; 95% CI: 1.29–1.47), use of non-invasive ventilation (OR: 1.14; 95% CI: 1.09–1.18), acute kidney injury (OR: 1.09; 95% CI: 1.04–1.13), sepsis (OR: 1.23; 95% CI: 1.10–1.37), and stroke (OR: 1.80; 95% CI: 1.40–2.32) [18]. It is also known that in a number of patients with COPD, pharmacological and electrical cardioversion for AF may not be effective until respiratory hypoxia is eliminated [19]. Thus, hypoxia is a risk factor for arrhythmias in patients with chronic obstructive pulmonary disease and heart failure [20]. Atrial fibrillation is associated with markers of hypoxia in atria [21].

### 3.2. Persistent Inflammation

Exposure to chronic hypoxia in combination with other insults leads to the development of oxidative stress, resulting in endothelial dysfunction and chronic inflammation, which has been investigated in a number of clinical studies. One study showed an increased number of CD45+ inflammatory cells in the tissues of both the left and right atria compared to the control group (*p* = 0.0018). At the same time, the number of inflammatory cells in the tissues of both atria correlated with each other (r = 0.6145; *p* = 0.0067), which may indicate chronic progressive inflammation [22].

In studies, the activity of the inflammatory process has most often been assessed based on the activities of biomarkers, most commonly based on the level of C-reactive protein (CRP). CRP is a serum protein—a marker of the acute phase of inflammation, involved in many immunoregulatory processes. It is known to activate the complement cascade, stimulating the process of the phagocytosis of opsonized bacterial cells by phagocytes. Most CRP is synthesized by the liver; however, some cells involved in the inflammatory response, such as certain populations of lymphocytes, monocytes, and Kupffer cells, can also produce CRP. CRP produced by alveolar macrophages is present in lung tissues and bronchoalveolar lavages [23]. 

In one study, the incidence of AF paroxysms was correlated with the amount of high-sensitivity CRP (r = 0.442; *p* = 0.001), as well as with echocardiographic parameters, such as the biventricular atrial size (*p* < 0.001), left ventricular IVRT (*p* = 0.022), and systolic blood pressure level in the pulmonary artery (PASP) (*p* < 0.001) [17]. In another study, the development of atrial fibrillation was also associated with higher levels of inflammation. For example, the group of patients with a CRP of > 3.41 mg/L was more likely to develop AF than the group with lower CRP levels (7.4% vs. 3.7%; OR: 1.8; 95% CI: 1.2–2.5; *p* = 0.002). Of the 5491 patients with a sinus rhythm enrolled in the study, 897 (16%) developed AF during the follow-up. The detection of an elevated CRP at the baseline was associated with a higher risk of future AF (OR: 1.31; 95% CI: 1.08–1.58; *p* = 0.005), and increasing CRP levels over time significantly predicted the development of AF in patients with a sinus rhythm (OR: 1.24; 95% CI: 1.11–1.40; *p* < 0.001) [24]. 

The association of inflammation with the likelihood of AF recurrence after successful cardioversion was evaluated in patients with nonvalvular AF who underwent electrical pulse therapy with the successful restoration of the sinus rhythm. The initial level of the CRP was determined in all the patients, and 4 groups, balanced for age, sex, ejection fraction, and left atrial size, were distinguished according to the level of the CRP. The patients with the lowest CRP levels (<1.9 mg/L) had a significantly lower rate of recurrent AF within one year than the other groups (*p* = 0.01), and an elevated CRP was associated with recurrent AF during the follow-up (OR: 4.98; 95% CI: 1.75–14.26; *p* = 0.003) [25]. 

A 2014 meta-analysis confirmed the role of CRP in predicting recurrent AF after successful cardioversion. CRP levels above the threshold were an independent predictor of AF recurrence after cardioversion (OR: 3.33; 95% CI: from 2.10 to 5.28). The sensitivity for elevated CRP was 71.0% (95% CI: from 63% to 78%), and the specificity was 72.0% (95% CI: from 61% to 81%). Most studies used a CRP threshold of 1.9 mg/dL to predict the long-term recurrence of AF (sensitivity 77%; specificity 65%) and 3 mg/dL to predict the short-term recurrence of AF (sensitivity 73%; specificity 71%) [26].

It is possible that chronic inflammation is a consequence, not a cause, of AF. This is the opinion of Josep M. Alegret et al., who studied the relationships between CRP and C-C motif chemokine 2 (CCL2) levels and the development of AF. Plasma concentrations of CRP and CCL2 were significantly higher in patients with AF than in those with a sinus rhythm; however, CRP was elevated only in patients with permanent AF, whereas CRP was normal in patients with paroxysmal AF. The authors concluded that elevated plasma CRP alone does not increase the risk of atrial fibrillation. CCL2 levels suggest that inflammation is probably a consequence of AF and depends on the duration of the arrhythmia [27]. 

The relationship between the presence in the blood of another systemic inflammatory factor, vascular cell adhesion molecule-1 (VCAM-1) levels, a marker of systemic endothelial damage, and the development of AF was investigated in patients with postoperative AF. Forty-four patients were followed for 72 h after elective coronary artery bypass graft surgery. Twenty-two percent of the patients developed postoperative AF, which was associated with elevated blood VCAM-1, an independent predictor of the development of postoperative AF [28]. Undoubtedly, chronic inflammation contributes to the activation of the hypothalamic–pituitary–adrenal axis with the release of endogenous glucocorticosteroids during COPD exacerbation, which may also enhance the processes of myocardial remodeling, leading to the development of AF [29].

The data on the role of macrophages in the pathogenesis of atrial fibrillation are of interest. Macrophages are active participants in the development and progression of COPD [30,31]. Their number is significantly increased in COPD due to the population of alveolar macrophages and the recruitment and differentiation of monocytes from the peripheral bloodstream. Macrophages are an integral part of a healthy working myocardium, including being associated with the cells of the cardiac conducting system [32]. Macrophages electrically modulate cardiomyocytes and influence conduction, as has been shown in the AV node [32]. The central role of recruited proinflammatory and profibrotic macrophages in the pathogenesis of atrial fibrillation has been demonstrated [33]. Immune remodeling involving macrophage recruitment and activation in atrial fibrillation is characterized by the release of proinflammatory factors, including tumor necrosis factor-α (TNF-α), macrophage migration inhibitory factor (MIF), interleukin-1-beta (IL-1β), IL-6, and galectin-3, which can induce atrial electrical remodeling [34]. Meanwhile, another study in a large cohort of postmenopausal women found no significant association between IL-1β and the development of AF, although downstream effectors, CRP and IL-6, were associated with the development of AF [35]. Another study showed that high levels of IL-6, IL-10, and TNF-α were associated with the progression of paroxysmal or chronic AF [36]. The importance of systemic inflammation was exemplified in a study in which lipopolysaccharide (LPS) induced the suppression of the L-type calcium-channel gene expression [37]. LPS stimulation promotes the macrophage production of IL-1β, which inhibits quaking protein (QKI) expression in atrial myocytes and further suppresses L-type calcium currents, which contribute to atrial electrical remodeling and exacerbate AF [38]. In addition, serum lipopolysaccharide has been found to be associated with atrial fibrillation recurrence after radiofrequency ablation by increasing systemic inflammation and atrial fibrosis [39].

Tobacco smoking is known to be associated with LPS-induced inflammation, which may be relevant to both COPD and AF. Tobacco smoking is a known key risk factor for COPD and AF. In a prospective study including more than 15,000 participants, smoking was shown to be associated with a high incidence of AF. The hazard ratios (HRs) for the development of AF were 1.32 (95% CI: 1.10–1.57) in former smokers, 2.05 (95% CI: 1.71–2.47) in current smokers, and 1.58 (95% CI: 1.35–1.85) in ever smokers [40]. Moreover, smoking cessation after AF diagnosis was associated with reduced risks of cardiovascular events, total stroke, and ischemic stroke [41]. 

Interestingly, in addition to immune cells, the activation of the NLRP3 inflammasome in atrial cardiomyocytes may be involved in the pathogenesis of atrial fibrillation [42]. These and other data strengthen the understanding of the links between COPD and AF involving inflammation and immune mechanisms.

Thus, both COPD and AF have immune mechanisms underlying their pathogenesis, many links of which are only beginning to be understood.

### 3.3. Endothelial Dysfunction

The damage to the vascular wall, caused by hypoxia and the toxic effects of tobacco smoke, leads to the development of endothelial dysfunction. Owing to the accumulation of free radicals, the protective properties of the endothelium are reduced, resulting in a decrease in nitric oxide synthesis owing to the suppression of the nitric oxide synthase function. The production of prostaglandins also decreases, and the role of damaging factors, including endothelin-2, thromboxane A2, and superoxide anions, increases. As a result of decreased nitric oxide production, the cell adhesion increases, the antiproliferative and antithrombotic properties of the endothelium are reduced, the sympathoadrenal system is activated, and vasoconstriction develops. The endothelium is damaged by free radicals, resulting in the increased production of interleukins 1 and 6 and the tumor necrosis factor. These processes activate cell adhesion with the infiltration of lymphocytes and monocytes into the subendothelial space, where monocytes, under the influence of endothelial differentiation factors, transform to macrophages and begin to secrete proinflammatory cytokines, chemoattractants, growth factors, and other biologically active substances that activate the myocytes of the vascular wall, as well as trap excess lipoproteins, transforming them to foam cells [43,44]. Endothelial damage due to chronic hypoxia and inflammation leads to the earlier onset of atherosclerosis in patients with COPD. Thus, in a study on mice that were modeled to develop atherosclerosis on a high-cholesterol diet, the atherosclerosis progressed faster in mice that were exposed to hypoxia and had higher low-density lipoprotein (LDL) values (124 ± 4 vs. 106 ± 6 mg/dL; *p* < 0.05) and was associated with a twofold increase in serum lipid peroxidation [45].

Endothelial dysfunction is common in patients with COPD, which is one of the mechanisms linking COPD and cardiovascular disease. In this regard, data on the association between endothelial dysfunction and AF are of interest. AF is an important cause of atrial and systemic endothelial dysfunctions, which are associated with several mechanisms [46]. On the other hand, endothelial dysfunction has been shown to be associated with an increased risk of atrial fibrillation [47,48,49]. Endothelial cells are involved in the control of atrial infiltration by immune cells and may be involved in myocardial fibrosis, oxidative stress, and inflammation [50]. 

### 3.4. Myocardial Remodeling

Increased myocardial stiffness is a natural consequence for remodeling processes occurring in patients with COPD. Thus, left ventricular diastolic dysfunction was detected in 88% of COPD patients (*p* < 0.05). Also in patients with a more severe course of COPD were more pronounced signs of right ventricular overload (*p* < 0.05) and dilatation (*p* < 0.05) [51]. In patients suffering from COPD, against the background of structural–functional changes in the myocardium, there are observed disturbances in the electrical systole of the left and right atria [52]. Despite the fact that the foci of the myocardial electrical inhomogeneity in AF are typically considered to be located at pulmonary vein orifices, the triggers of the atrial fibrillation paroxysm can be foci localized in the right atria. Thus, when analyzing the atrial electrophysiological study of 45 patients with frequent episodes of atrial fibrillation (mean duration: 344 ± 326 min per day), 3 foci (6.66%) were found in the right atrium, and 16 patients (35.56%) had 2 or more points of ectopic activity [53]. 

A study conducted in 2015 was designed to identify the features of echocardiographic parameters in 94 patients with stable COPD and paroxysmal atrial fibrillation. Of the 94 patients included in the study, paroxysms of AF, according to the data of daily ECG monitoring, were registered in 46 subjects, and in 22 patients (47.8%), AF was detected for the first time. The echocardiography parameters, including the left atrial volume index (LAVI), left ventricular IVRT, systolic blood pressure level in the pulmonary artery, basal right ventricular diameter, and right atrial area, were statistically significantly correlated with the number of AF paroxysms per day. The correlations of the FEV1 with the right atrial area, right ventricle size, systolic blood pressure level in the pulmonary artery, LAVI, and IVRT were also noted [17].

As pulmonary hypertension progresses, adaptive mechanisms become impaired, leading to the development of chronic pulmonary heart disease. Pulmonary heart disease is characterized by right ventricular myocardial hypertrophy and impaired diastolic and then systolic functions of the right ventricle, with the subsequent dilatation of the right ventricle. Factors such as the vasoconstriction of pulmonary arterioles in response to hypoxia; the destruction and remodeling of pulmonary vessels, with the development of endothelial dysfunction; the imbalance in vasoactive substances, including a decrease in endogenous nitric oxide and an increase in endothelin-1; and the development of compensatory erythremia contribute significantly to the pathogenesis of chronic pulmonary heart disease [54]. The association of increased right heart strain with the development of AF was evaluated in a study of 2246 patients with preserved left ventricular systolic and diastolic functions (left ventricular ejection fraction >50%; left atrium volume index <34 mL/m^2^; E/e′ ratio <14). The risk of AF was significantly higher with increased pulmonary artery systolic pressure regardless of the left ventricular structure and function, heart rate, body mass index, presence of sleep apnea, blood pressure (BP) levels, antihypertensive medication use, and pulmonary, kidney, and thyroid functions (OR: 1.65; 95% CI: 1.08–2.54 for a tricuspid regurgitation jet velocity of >2.8 m/s) [55]. 

Increased right heart strain may lead to impaired LV diastolic function. A study to evaluate the role of increased pulmonary artery pressure (PAP) in the development of left ventricular diastolic dysfunction included 2 groups of patients with 22 participants each. The main group included patients with COPD, and the control group included patients without COPD, with a normal LV ejection fraction, according to echocardiography, and without CHD. All the patients underwent transthoracic echocardiography; 13 patients with COPD had their PAP measured using invasive techniques. The ratio of the early peak flow velocity to the late peak flow velocity (E/A) was significantly reduced in COPD patients compared to controls (0.79 ± 0.035 vs. 1.38 ± 0.069; *p* < 0.0001), indicating an impaired left ventricular diastolic function. The atrial contribution to the total left ventricular diastolic filling was higher in patients with COPD, even in patients with a normal PAP, as determined by invasive monitoring. PAP was correlated with the E/A ratio in this study (r = −0.85; *p* < 0.0001). The authors of this study concluded that left ventricular diastolic dysfunction is present in COPD patients with a normal PAP and is exacerbated by right ventricular overload [56].

Structural changes in the myocardium of COPD patients may lead to the appearance of arrhythmogenic foci due to changes in the geometry of the heart and vessels against the background of the progression of bronchopulmonary pathology. Thus, in a study by Roh, S.Y. on 15 patients with chronic lung diseases (8 patients—COPD; 6—tuberculosis-destroyed lung; 1—interstitial lung disease), changes in the morphology of the pulmonary veins, including their obliteration, and in the case of a unilateral destructive process, the displacement of pulmonary veins toward the destroyed lung or the compensatory bulging of the anterior part of the LA, were frequently revealed. These changes were associated with arrhythmogenicity in 40% of the patients. The patients with chronic bronchopulmonary disease were also more likely to have extrapulmonary foci of arrhythmogenic activity in the right atrium (26.7% vs. 5.0%; *p* = 0.025). After successful radiofrequency ablation (RFA), the rate of recurrent AF was slightly higher in the chronic bronchopulmonary disease group than in the control group but did not reach statistical significance (26.7% vs. 18.3%; *p* = 0.45) [57].

The mechanisms of atrial flutter (AFL) development in patients with COPD and the efficacy of RFA were investigated in another study. Of 181 patients who underwent percutaneous ablation for AF, 28 (15.5%) had COPD. Typical AFL was more common in the COPD group (68% vs. 33%; *p* = 0.006), and the prevalence of AFL increased with increasing COPD severity: 4 (50%) of 8 patients with mild COPD (GOLD 1), in 13 (72%) of 18 patients with moderate COPD (GOLD 2), and in 2 (100%) of 2 patients with severe COPD (GOLD 3). The atrial flutter cycle’s length and conduction time from the coronary sinus ostium to the low lateral right atrium during coronary sinus pacing before and after cavotricuspid isthmus ablation were significantly longer in the COPD group than in the group without COPD (285 vs. 236, 71 vs. 53, and 164 vs. 134 ms; *p* = 0.009, 0.03, and 0.002, respectively) [58].

## 4. Effects of Medications Used to Treat COPD

Modern COPD therapy includes a number of drugs with different mechanisms of action. Among the drugs that directly or indirectly affect the cardiovascular system, short- and long-acting β2-adrenoceptor agonists, muscarinic antagonists (antimuscarinics), topical and systemic glucocorticosteroids (GCSs), and methylxanthines are widely used. 

The adverse effects of β2-adrenoceptor agonists and antimuscarinics are probably due to their mechanisms of action and are realized through an increase in the heart rate (HR). The long-term use of β2-adrenergic agonists has been shown to increase the risks of myocardial infarction, congestive heart failure, cardiac arrest, and sudden cardiac death. For example, according to a meta-analysis of 50 studies, the use of a single β2-adrenergic receptor agonist increased the heart rate by 9.12 beats per minute (95% CI: 5.32–12.92) and decreased the potassium concentration by 0.36 mmol/L (95% CI: 0.18–0.54) compared to a placebo. Long-term β2-adrenergic receptor agonist therapy also significantly increased the risk of cardiovascular events compared to a placebo (OR: 2.54; 95% CI: 1.59–4.05) [59]. Nevertheless, the risk of arrhythmias is low with modern long-acting β2-adrenergic receptor agonists. A study by Hanrahan, J.P. analyzed the results of daily ECG monitoring in 1429 patients. Groups receiving long-acting β2-adrenergic receptor agonists at different doses or a placebo were compared. ECG monitoring was performed before the treatment and on the first day of the drug’s administration and then at week 6 and week 12. Four types of arrhythmias were evaluated: atrial tachycardia, atrial fibrillation/flutter, “nonsustained” (4–10 complexes according to the ECG) ventricular tachycardia, and “sustained” (more than 10 complexes according to the ECG) ventricular tachycardia. The proportion of the patients with atrial tachycardia occurring during the treatment ranged from 27% to 32% and was not significantly different in the β2-adrenergic receptor blocker groups compared with the placebo group (*p* = 0.70). There were also no significant differences between the groups for other arrhythmias. In addition, the use of long-acting β2-agonists did not increase the mean heart rate in any of the groups, regardless of the drug dose [60].

The leveling of the negative effects of β2-agonists on the cardiovascular system can be achieved by prescribing β-adrenoblockers, drugs that are used unreasonably infrequently in COPD patients [11]. A retrospective cohort study using the NHS Tayside Respiratory Disease Information System (TARDIS), conducted in Scotland between 2001 and 2010, including 5977 patients aged >50 years and diagnosed with COPD, with a median follow-up of 4.35 years, showed that the use of beta-blockers reduced all-cause mortality by 22% (OR: 0.28; 95% CI: 0.21–0.39) for treatment with inhaled corticosteroids (ICSs), long-acting beta-2-agonists, and long-acting antimuscarinics compared to treatment without beta-blocker use [61]. There are also data available on the positive effects of groups of drugs, such as statins, angiotensin-converting enzyme inhibitors (ACEIs), and glucocorticosteroids, on the prevention of AF recurrences. Perhaps the effectiveness of the latter is due to their influences on the mechanisms of the systemic inflammation [62].

Despite a number of adverse effects on the cardiovascular system, aminophylline is still widely used in COPD therapy. The ability of aminophylline to increase the HR can be used in patients with AF with a slow ventricular response [63]. However, in patients with a sinus rhythm, the use of aminophylline may provoke paroxysmal AF, even at a low risk of CVD [64].

The effects of glucocorticosteroids on the development of AF in patients with COPD are ambiguous. Thus, the use of systemic glucocorticosteroids leads to an increase in the BP—one of the main risk factors for the development of AF. At the same time, glucocorticosteroids reduce systemic inflammation, which also affects the development of AF. The use of inhaled glucocorticoids (IGCs) seems to be promising in this case. Thus, in a study conducted on 41 patients with mild or moderate COPD, the effects of systemic and inhaled glucocorticosteroids were compared with the effects of a placebo on the CRP level. The withdrawal of the inhaled glucocorticosteroid increased CRP levels by 71% (95% CI: 16–152%). The administration of the inhaled glucocorticosteroid (fluticasone) reduced CRP levels by 50% (95% CI: 9–73%), and prednisolone reduced them by 63% (95% CI: 29–81%). Taking the placebo had no effect on the CRP levels. Resuming fluticasone after its temporary withdrawal resulted in a 29% (95% CI: 7–46%) reduction in CRP. The authors concluded that inhaled and oral corticosteroids are effective in reducing serum CRP levels in patients with COPD and suggested their potential use in improving cardiovascular outcomes in COPD [64]. 

Statins, which are widely used in the therapy of CVDs, also have anti-inflammatory effects, and statin therapy may influence cardiovascular outcomes in COPD patients. The anti-inflammatory effects of statins are described in a review by Hothersall, E. Probably, statins reduce the stability of the lipid raft formation, with subsequent effects on immune activation and regulation, as well as prevent the prenylation of signaling molecules with the subsequent suppression of the gene expression. The realization of these mechanisms lead to decreases in the expressions of cytokines, chemokines, and adhesion molecules, which have an effect on apoptosis and cell proliferation [65].

## 5. Modern Methods of Early Detection and Prognosis of Atrial Fibrillation in Patients with COPD

Currently, there are a number of models that enable the prediction of the development of AF in patients with a sinus rhythm. At the same time, the issues for predicting the development of AF in patients with COPD remain largely open. Some prognostic scales include COPD as one of the risk factors for AF development. To determine the risk of AF development in patients with COPD, it is possible to analyze the levels of some biomarkers, as well as ECG and echocardiographic features. One of the promising directions in predicting AF is the use of machine-learning methods. It should be noted that to date, the ideal model of AF prediction has not been developed, and the listed directions of AF prediction are developing and improving in parallel. The most interesting methods of early AF detection that can be applied to COPD patients will be discussed below.

### 5.1. Prediction of Atrial Fibrillation Based on Biomarkers

O’Neal, W.T. et al. described the possibilities for predicting AF development on the basis of various biomarkers. B-type natriuretic peptide (BNP) and the stable N-terminal part of the prohormone pro-BNP (NT-pro-BNP), which are peptides synthesized by cardiomyocytes in response to increased pressure and resulting in myocardial stretching, were proposed [66]. A number of studies have demonstrated that NT-pro-BNP is significantly associated with the occurrence of AF events. For example, the Framingham Study showed that BNP could predict the occurrence of AF, and this marker improved the predictive ability of the AF risk scale developed in that study.

Interestingly, the NT-proBNP level is an independent predictor of COPD exacerbation, even in individuals without overt heart disease [67]. In addition, NT-proBNP levels were higher in COPD patients with an FEV1 of <50% compared with patients with higher FEV1 values [68]. In general, elevated NT-proBNP levels are a marker of a more severe course of COPD with worse clinical outcomes [68]. The development and progression of AF (from paroxysmal to persistent) are associated with gradual increases in serum levels of NT-proBNP and IL-6 and the MMP-9/TIMP-1 ratio [69]. 

CRP, often elevated in COPD exacerbation, is also the most widely studied proinflammatory marker of AF risk, although the addition of this inflammatory marker did not improve the predictive ability of the Framingham Study’s scale. 

### 5.2. Prediction of Atrial Fibrillation Based on Functional Tests

ECGs are an accessible and inexpensive method to assess cardiac rhythm and conduction abnormalities. Attempts to predict the occurrence of AF via ECGs have been made repeatedly, and in some cases, have been quite successful. In patients with COPD, owing to the increased load on the right and then the left atria, ECG changes can be a good way to detect AF early. 

The P-waveform in an electrocardiogram (ECG) measured at rest with 12 leads is an indication of the atrial electrophysiology. It is believed that P-waveform changes represent a delay in atrial depolarization resulting from fibrosis, dilatation, and increased filling pressure. Thus, an increase in the amplitude of the P-tooth in an ECG at the V1 lead is associated with the occurrence of AF. The duration of the P-waveform was a predictor of AF occurrence in the Framingham Study. The relationship between the P-wave’s duration and AF was nonlinear; the shortening of the P-wave duration was also a predictor of AF occurrence. A prolonged PR interval was also included in the risk assessment of AF in the Framingham Study [70]. The ECG’s P-wave dispersion, calculated as the difference between the minimum and maximum P-wave durations, is considered as one of the predictors of AF development. Dilaveris, P.E. et al. investigated ECG P-waveform dispersions for 88 patients aged 64 ± 12 years and with paroxysmal symptomatic AF. The maximum P-wave duration and P-wave variance were calculated from the surface ECGs recorded using 12 leads for all the patients during the sinus rhythm. The maximum ECG P-wave duration (*p* < 0.001) and age (*p* = 0.037) were found to be independent predictors of frequent AF paroxysms [71]. 

Echocardiographic examination, especially the assessment of the atrial structure and function, is also an available tool for predicting the development of AF. Thus, in a study by De Vos, C.B., tissue Doppler echocardiography was used to predict the atrial conduction time as the time from the initiation of the P-wave in the ECG (lead II) to the A′ wave in the lateral left atrial tissue’s Doppler imaging (PA-TDI). A total of 249 patients with no history of AF were included in the study. During a follow-up period of 1.86 ± 0.79 years, AF developed in 15 patients (6%). These patients had a longer PA-TDI interval than patients with a sinus rhythm (172 ± 25 ms vs. 150 ± 20 ms; *p* = 0.001). After adjusting for potential factors, the Cox regression showed that the PA-TDI score was independently associated with first-time AF (OR: 1.375; 95% CI: 1.037–1.823; *p* = 0.027). The incidence of AF development within 2 years was 33% in patients with a PA-TDI interval of >190 ms compared with 0% in patients with a PA-TDI interval of <130 ms (*p* = 0.002) [72]. A successful attempt to predict the development of AF based on echocardiography was made by Xu, H.F. et al. In 2011, they described the dependence of AF paroxysm occurrences on the thickness of the posterior wall of the left ventricle. The study included 2 groups of patients with 166 participants in each group. The main group included patients with paroxysmal atrial fibrillation; the control group included patients without cardiovascular diseases. The groups were comparable by sex and age. A multivariate analysis showed that the left ventricular posterior wall’s thickness, tricuspid regurgitation, and left atrial diameter were independent predictors of paroxysmal atrial fibrillation [73]. In another study, Hirose, T. et al. evaluated the left atrium in 580 patients with echocardiograms in the apical four-chamber position. The mean age of the patients was 64 ± 17 years, and the patients had no history of AF. The follow-up period was 28 months. During the follow-up period, AF developed in 32 patients (5.52%; mean age: 73 ± 9 years; 56.25% male). The group of patients with the developed AF differed significantly in the left atrial ejection fraction (16 ± 5% vs. 28 ± 8%; *p* < 0.001), myocardial strain rate (−0.9 ± 0.2 vs. −1.4 ± 0.5; *p* < 0.001), and left atrium volume index (59 ± 12 vs. 46 ± 16 mL/m^2^; *p* < 0.001). In a multivariate logistic regression analysis, a left atrial ejection fraction of less than 20% was an independent predictor of first-time AF [74].

Computed tomography (CT) is widely used in the diagnosis of both pulmonary and cardiovascular pathologies. The results of the CT of the left atrium after radiofrequency ablation (RFA) were the reason for creating a prognostic model. The study included 260 patients after ablation for AF and 30 control patients without a history of AF. All the patients with AF underwent 30-day ECG monitoring and a cardiac CT scan 3–4 months after the ablation procedure. The correlations between the anatomical and functional parameters of the left atrium and the development of AF recurrences were obtained, and AF paroxysms were detected by monitoring in 26.9% of the patients [75]. In another study, the left atrial wall’s thickness was measured using CT. A reduced left atrial wall thickness was an independent predictor of paroxysmal AF (AUC: 0.706; sensitivity: 62%; specificity: 73%) [76].

The use of magnetic resonance imaging (MRI) of the left atrial wall in conjunction with complex fractionated atrial electrograms (CFAEs) may also be a powerful tool for predicting the development of AF. In a study by Hwang, S.H., the substrates of the left atrial wall were categorized based on MRI findings as fibrotic, intermediate, and normal. The composition ratio was calculated as a percentage of the volume of the left atrial wall’s area. The content of the normal substrate was significantly higher in a left atrial wall with a normal electrical activity (52 ± 38% vs. 20 ± 28%; *p* < 0.01), and there was a lower content of the fibrotic substrate (7 ± 17% vs. 21 ± 24%; *p* < 0.01) [77].

### 5.3. Application of Multifactorial Clinical Models

Although multifactorial prognostic models are widely used in predicting AF, no specific models have been developed for the early detection of AF in COPD patients. However, some scales include COPD as a risk factor for AF, but these are not without their limitations. For example, as the number of analyzed variables increases, the contribution of each of them to the development of the disease decreases. The most difficult stage for building a multifactorial model is the identification of independent predictors of the pathological process’s development [78]. 

One of the first successful developments of a score for predicting the development of AF was conducted within the framework of the Framingham Study (FHS). From 8044 patients (mean age: 60.9 years), 4764 participants aged 45–95 years were selected. Initially, about 5% of the subjects had ECG changes in the form of evidence of left ventricular hypertrophy (LVH), CHF, or a history of myocardial infarction (MI). During 10 years, 457 subjects (9.99%) developed AF. There were totals of 6.3 per 1000 person-years of events in men and 3.3 per 1000 person-years of events in women. Factors such as age; sex; body mass index (BMI); blood pressure (systolic, pulse pressure, and therapy taken for AH); electrocardiographic features, including left ventricular hypertrophy and PR interval duration; prevalence of cardiovascular disease, including heart failure and myocardial infarction; and presence of a heart murmur influenced the development of AF. The final model showed a C-statistic = 0.78 (95% CI: 0.76–0.80; *p* < 0.05). The 10-year AF risk varied by age: a 10-year AF risk of >15% was observed in 1.0% of those younger than 65 years compared with 26.9% of the participants older than 65 years. The inclusion of echocardiogram measurements in the model marginally improved the C-statistic to 0.79 (95% CI: 0.77–0.82; *p* = 0.005) (Table 1) [79].

Another method for predicting the development of AF based on clinical data was presented by Chamberlain, A.M. et al. in the form of a 10-year prediction scale for first-onset AF. The scale is based on data from 14,546 participants in the ARIC study. During 10 years of follow-up, 515 cases of AF development were identified. The predictive model was based on parameters such as age, race, height, smoking status, systolic blood pressure, antihypertensive medication, presence of heart murmurs, left ventricular hypertrophy, left atrial enlargement, diabetes mellitus, ischemic heart disease, and chronic heart failure. The model showed good predictive validity, with an AUC of 0.77 (95% CI: 0.75–0.78; *p* < 0.01), which was significantly higher than that in the Framingham Study [80].

Alonso, A. et al. proposed a model based on three large US registries: the ARIC (Atherosclerosis Risk in Communities study), CHS (Cardiovascular Health Study), and FHS (Framingham Heart Study). The CHARGE-AF model included data from 18,556 patients aged from 46 to 94 years, totaling 1186 cases of AF. The validation of the model was performed on 7672 participants in the AGES (Age, Gene, and Environment-Reykjavik Study) and RS (Rotterdam Study), with 585 cases of AF. The 5-year prognostic model included variables such as age, race, height, weight, systolic and diastolic blood pressures, smoking status at the time of the study, antihypertensive medication use, diabetes mellitus, history of myocardial infarction, and heart failure. The model showed good predictive validity (C-statistic: 0.765; 95% CI: 0.748–0.781). Adding ECG measurements to the model did not change the predictive power of the model. The model’s validation results in the validation cohorts were worse (AGES C-statistic: 0.664; 95% CI: 0.632–0.697 and AUC RS C-statistic: 0.705; 95% CI: from 0.664 to 0.747) [84].

In further studies, the number of participants continued to grow. For example, in developing a validated HATCH scale, data from 670,804 patients over 20 years of age and without a history of cardiac rhythm disturbances were used. The scale represents a risk score (hypertension: 1 point; age > 75 years: 1 point; stroke or transient ischemic attack: 2 points; chronic obstructive pulmonary disease: 1 point; heart failure: 2 points) and was originally proposed to predict the progression of AF from the paroxysmal to the permanent form. The study evaluated the Taiwan National Health Insurance Research Database. The mean age of the participants was 42.4 ± 16.0 years, and the majority of the patients (89.4%) had a HATCH score of 0. The follow-up period was 9.0 ± 2.2 years. AF developed in 9174 (1.4%) patients, and the incidence of AF was 1.5 per 1000 patient-years. The incidence increased from 0.8 per 1000 patient-years for patients with a HATCH score of 0 to 57.3 per 1000 patient-years for patients with a HATCH score of 7. The incremental risk for developing AF increased by 2.059 (95% CI: 2.027–2.093; *p* < 0.001) for each additional score on this scale. The performance of the predictive model was high: C-statistic = 0.716 (95% CI = 0.710–0.723; *p* < 0.001). It is worth noting that the HATCH scale was the first to evaluate the presence of COPD in a patient as one of the criteria for a high probability for developing AF [83]. 

The C2HEST model was developed by analyzing the case histories of 471,446 patients in the Chinese Yunnan Insurance Database and tested on 451,199 patients in the Korean National Health Insurance Service. Structural heart disease, heart failure, age ≥75 years, CHD, thyrotoxicosis, COPD, and arterial hypertension were used to develop the scale. Patients with structural heart disease were excluded from the study because of their initial high risk for developing AF (OR: 26.07; 95% CI: 18.22–37.30; *p* < 0.001). The efficacy of the model was quite high (AUC = 0.75; 95% CI: 0.73–0.77; *p* = 0.774) [82].

The CHARGE-AF model for predicting the 5-year risk of AF was evaluated using a primary care database in the Netherlands (“Nivel-PCD”). A total of 111,475 patients without a history of AF were included in the study. The CHARGE-AF model includes parameters such as age, race, height, weight, smoking status, systolic and diastolic BPs, medication intake in relation to arterial hypertension, diabetes mellitus, histories of myocardial infarction and CHF, signs of left ventricular hypertrophy, and prolongation of the PR interval in ECGs. The mean age of the subjects was 65.5 years, and the mean follow-up period in the sample was 3.5 ± 1.7 years. There were 5264 (4.7%) new cases of AF during the follow-up period. The CHARGE-AF C-statistic for the occurrence of AF was 0.74 (95% CI: from 0.73 to 0.74) [81]. 

It is worth noting that the presence of COPD as a risk factor for AF was included in only two validated scales with high performance (Table 2). Three scales include smoking status as a major risk factor for COPD. None of the above clinical scales has been validated in patients with chronic obstructive pulmonary disease, and their efficacy in this cohort of patients is unknown. It should be noted that specific multifactorial scales to predict the development of AF in patients with COPD have not been found in the literature available to the authors.

### 5.4. Application of Machine-Learning Methods

With the spread of electronic medical records and the accumulation of large amounts of data, new tools for analyzing and predicting clinical outcomes have emerged. The main tasks of such data analyses are the standardization and integration of disparate research results into a unified system [85]. 

Artificial intelligence is also becoming increasingly important in biomedical research, primarily in the diagnosis and prediction of clinical outcomes, for which one of the promising areas of development is the use of machine learning (ML). Thus, Koohy, H. analyzed publications in PubMed from 1990 to 2017 that mentioned ML, showing a significant increase in interest in its methods [86]. Unlike standard linear and logistic regression analysis tasks, which require the identification of sufficiently accurate and significant relationships to build a regression model, ML enables us to solve classification (clustering) problems based on the probabilities for belonging to a particular class. The main algorithm for solving classification problems in ML is the creation and training of neural networks (NNs). The general principles of the neural network (NN) architecture can be presented as follows: All NNs have an input layer to which trained data are fed. The data should be vectors of values, usually normalized between 0 and 1 or from −1 to 1. The input layer is followed by hidden layers, where the information is processed. Each successive layer accepts the information processed by the previous layer of neurons. The output layer is a classifier, which determines, using one of the algorithms, to which class the object belongs [87].

Currently, machine-learning methods and trained neural networks are widely used to detect and predict clinical outcomes in AF [88]. 

Tiwari, P.; Colborn, K.L.; Smith, D.E.; Xing, F.; Ghosh, D.; and Rosenberg, M.A. developed a machine-learning model based on data from patients’ electronic medical records and the Observational Medical Outcomes Partnership Common Data Model for identifying the risk of AF (Table 3). This processed 2,252,219 electronic medical records of patients treated between 2011 and 2018. Of the 2,252,219 participants (1,225,533 (54.4%) women; mean age: 42.9 ± 22.3 years), 28,036 (1.2%) had AF diagnosed within 6 months. The model took 200 parameters as input and consisted of a single multilayer perceptron (MLP). The resulting model was comparable in performance to existing clinical scales based on logistic regression (AUC = 0.80; F1 score: 0.11) [89]. 

A similar machine-learning algorithm was used in a study by Sekelj, S. et al. The DISCOVER registry was used to test the neural network, and the study included data from 2,542,732 patients aged over 30 years and without a diagnosis of atrial fibrillation during the previous 5 years. A separate subgroup analysis of patients aged >65 years was performed. The algorithm classified 604,135 patients (23.8%) as at risk for developing AF within 6 months. Of these, AF was detected in 3.0% (17,880 patients) before the end of the study. The AUC was 0.83, with a method sensitivity of 75.0% and a specificity of 99.1%. Among the 117,965 patients older than 65 years of age, the sensitivity was 91.8%, and the specificity was 96.7% [90].

Hill, N.R. et al. compared previously used prognostic scales (Framingham, ARIC, and CHARGE-AF) and a new machine-learning model for predicting AF based on the addition and updating of patients’ information over time. A total of 2,994,837 patients were included in the analysis, and 3.2% developed AF during the study. The AUC was 0.827 versus 0.725, with a sensitivity of 75%, significantly exceeding the best CHARGE-AF model accepted into the study [91].

Functional tests and imaging techniques have become a wide field for modeling and training neural networks, especially with the development of digital processing and storage techniques. Electrocardiography remains the most common method for diagnosing cardiovascular diseases. An electrocardiograph supported by artificial intelligence, which was a convolutional neural network (CNN), was presented at the Mayo Clinic’s ECG laboratory. The neural network detected patterns characteristic of AF in 10 s sinus-rhythm ECGs. A total of 180,922 patients and 649,931 ECGs were included in the analysis, and 3051 patients (8.4%) had confirmed AF. The technique was quite effective, with an AUC of 0.87 (95% CI: from 0.86 to 0.88), and the method’s sensitivity was 79.0% (95% CI: from 77.5 to 80.4) and specificity was 79.5% (95% CI: from 79.0 to 79.9). The F1 criterion was 39.2% (from 38.1 to 40.3), and the overall accuracy was 79.4% (from 79.0 to 79.9) [92]. Another neural network was built by analyzing the heart rate variability. In that study, 30 min ECG recordings preceding AF paroxysm were analyzed. The sensitivity of the method was 100%, the specificity was 95.55%, and the accuracy was 98.21% [93].

Firouznia, M. et al. developed a machine-learning algorithm based on cardiac CT scans that was able to predict AF recurrence after ablation, with an AUC of 0.87 [94]. An original NS that could predict AF recurrence was also developed based on the analysis of left atrial CT images. Sixty-eight patients with AF who underwent cardiac CT before the ablation procedure were included in the study. Differences in the left atrial shape between patients with and without AF recurrence were identified, and based on this, an NS was constructed and trained. The performance in predicting AF recurrence was AUC = 0.67 for CT findings and AUC = 0.78 when clinical methods were combined with the NS performance [95].

No studies of the effectiveness for using machine-learning methods in predicting AF in patients with COPD were found in the literature available to the authors.

## 6. Conclusions

Atrial fibrillation and COPD often occur in the same patient, which is of great importance for the courses of both diseases and affects their prognoses. A growing body of evidence suggests that these diseases have multiple links, including immune mechanisms and systemic inflammation, endothelial dysfunction, and myocardial remodeling processes. These processes usually occur over a long period of time and are a part of the natural course of COPD. In this regard, tools that enable the prediction of the development of AF to correct known risk factors are of clinical interest. Currently, several prediction models are known that are based on the analysis of several clinical parameters, including smoking status and the presence of COPD. At the same time, no model that is focused on predicting AF in patients with COPD has been proposed so far. On the other hand, it should be noted that the simple consideration of COPD as a single factor does not fully correspond to the modern understanding of the natural course of COPD, which implies the progression of the disease, including a decline in the pulmonary function and the development of pulmonary and extrapulmonary manifestations. In this regard, the assessment of clinical and functional characteristics indicating the course of COPD is of clinical interest as potential markers in prognostic models.

Thus, the combination of COPD and AF is an important medical problem; the keys to solving it are currently unknown to clinicians and researchers. The development of new prognostic models can improve the management of patients with COPD and improve the quality and efficiency of medical care.

## Figures and Tables

**Table 1:** Comparison of the performances of the predictive multivariate models.

Study	Number of Participants	Performance Indicator in the Study ^1^	Reference
FHS	49,599	AUC = 0.78 (0.76–0.80)	[79]
ARIC	14,546	AUC = 0.77 (0.75–0.78)	[80]
CHARGE-AF	18,556	AUC = 0.77 (0.75–0.78)	[81]
C2HEST	471,446	AUC = 0.75 (0.73–0.77)	[82]
HATCH	670,804	AUC = 0.72 (0.71–0.72)	[83]

^1^ Performance indicators are given in the format of the area under the ROC curve and a 95% confidence interval.

**Table 2:** AF risk factors used in prognostic models.

Factor	FHS	ARIC *	CHARGE-AF **	C2HEST **	HATCH *
Blood pressure	+	+	+	+	+
Weight			+		+
Age	+	+	+	+	+
Left Ventricular Hypertrophy		+			
Ischemic Heart Disease		+		+	
Stroke or Transient Ischemic Attack					+
History of Myocardial Infarction	+		+		+
Body Mass Index	+				
Smoking Status		+	+		+
Sex	+				
Race		+	+		+
Height		+	+		+
Diabetes Mellitus		+	+		+
Heart Murmur	+	+			
Thyrotoxicosis				+	
Left Atrial Enlargement		+			
COPD			+	+	
Chronic Heart Failure	+	+	+	+	+
Electrocardiogram	+				+

Note: *—includes COPD risk factors; **—includes COPD.

**Table 3:** Comparison of the effectiveness of machine-learning models.

Study	Number of Participants	Performance Indicator in the Study ^1^	Reference
Tiwari, P. et al.	2,252,219	AUC = 0.80	[89]
Sekelj, S. et al.	2,542,732	AUC = 0.83	[90]
Hill, N.R. et al.	2,994,837	AUC = 0.83	[91]

^1^ Performance indicators are given in the format of the area under the ROC curve.

## Data Availability

Not applicable.
